# Critical Molecular and Cellular Contributors to Tau Pathology

**DOI:** 10.3390/biomedicines9020190

**Published:** 2021-02-14

**Authors:** Liqing Song, Evan A. Wells, Anne Skaja Robinson

**Affiliations:** Department of Chemical Engineering, Carnegie Mellon University, Pittsburgh, PA 15213, USA; liqing@cmu.edu (L.S.); eawells@andrew.cmu.edu (E.A.W.)

**Keywords:** tauopathies, Alzheimer’s disease, prion-like propagation, tau self-aggregation, endocytosis, neuron-glial communication, neuroinflammation, apolipoprotein E

## Abstract

Tauopathies represent a group of neurodegenerative diseases including Alzheimer’s disease (AD) that are characterized by the deposition of filamentous tau aggregates in the brain. The pathogenesis of tauopathies starts from the formation of toxic ‘tau seeds’ from hyperphosphorylated tau monomers. The presence of specific phosphorylation sites and heat shock protein 90 facilitates soluble tau protein aggregation. Transcellular propagation of pathogenic tau into synaptically connected neuronal cells or adjacent glial cells via receptor-mediated endocytosis facilitate disease spread through the brain. While neuroprotective effects of glial cells—including phagocytotic microglial and astroglial phenotypes—have been observed at the early stage of neurodegeneration, dysfunctional neuronal-glial cellular communication results in a series of further pathological consequences as the disease progresses, including abnormal axonal transport, synaptic degeneration, and neuronal loss, accompanied by a pro-inflammatory microenvironment. Additionally, the discovery of microtubule-associated protein tau (*MAPT*) gene mutations and the strongest genetic risk factor of tauopathies—an increase in the presence of the ε2 allele of apolipoprotein E (*ApoE*)—provide important clues to understanding tau pathology progression. In this review, we describe the crucial signaling pathways and diverse cellular contributors to the progression of tauopathies. A systematic understanding of disease pathogenesis provides novel insights into therapeutic targets within altered signaling pathways and is of great significance for discovering effective treatments for tauopathies.

## 1. Introduction

Intraneuronal accumulation of neurofibrillary tangles (NFT) made of abnormally hyperphosphorylated tau is centrally involved in the pathogenesis of primary tauopathies, such as supranuclear palsy (PSP), corticobasal degeneration (CBD), Pick’s disease (PiD), and frontotemporal dementia with Parkinsonism linked to chromosome 17 (FTDP-17), and secondary tauopathies such as Alzheimer’s disease (AD) [1]. The development of tau pathology has been postulated to follow spatiotemporal patterns, starting from the dissociation of phosphorylated tau from microtubules and followed by the formation of toxic tau species via self-aggregation [2]. Even though polyanionic molecules are normally required for inducing tau aggregation in vitro, modifications to tau, such as site-specific mutations and site-specific phosphorylation, have driven spontaneous seeding and self-aggregation of tau in vivo under pathological situation [3]. Physiologically, extracellular tau is present in brain interstitial fluid (ISF) and then passes into the cerebrospinal fluid (CSF) [4,5]; however, the elevated concentrations of tau found in the brain ISF of human P301S tau transgenic mice has suggested that cellular tau release may be a part of disease progression [6]. Additionally, soluble tau concentrations in brain homogenates decrease with the deposition of intracellular insoluble tau, suggesting that transcellular tau propagation requires cellular internalization of extracellular tau, which has also been found to mediate the progression of neurodegeneration [6,7,8]. The cellular pathways for internalizing tau species are regulated by both heparan sulfate proteoglycan (HSPG)-mediated cellular uptake and specific receptor-mediated endocytosis, which are highly dependent on the isoform being internalized [8,9,10].

Extensive experimental data have demonstrated that transcellular propagation of soluble tau species occurs mainly through synaptic connections, leading to neuronal dysfunction characterized by the breakdown of cytoskeletal integrity, abnormal axonal transport, and synapse loss [9,10]. In particular, glial cells, activated microglia, and reactive astrocytes are also involved in the progression of tau pathology by directly affecting the homeostasis of the neuronal microenvironment or indirectly exerting inflammatory effects across multiple tauopathies [11,12]. For example, the degree of glial cell activation correlates with the severity of neurodegeneration in AD, in terms of the degeneration of synapses, neuronal loss, the formation of NFTs, or even cognitive impairment [13]. Alternatively, dysfunctional neuron-glial communication has been widely observed in AD patients and has recently developed in vitro tau pathology animal models [14,15]. Abnormal neuron-glial crosstalk strongly impairs neuronal homeostasis including neuronal metabolism, synaptogenesis, neurotransmission, and neuromodulation, contributing to the progression of neurodegeneration [14,16]. The investigation of critical molecular and cellular contributors to tau pathology provides a comprehensive understanding of tau pathogenesis that will accelerate the discovery of novel therapeutic targets and the development of drugs for treating tauopathies.

The purpose of this review is to summarize the factors that contribute to the formation of tau aggregates, tau cell-to-cell propagation, and glial contributions in tauopathies, by using the scientific evidence published in the last decade that bring promising insights into the therapeutic development for tau protein pathology. Keywords for this topic, such as tauopathies, Alzheimer’s disease, prion-like propagation, tau self-aggregation, endocytosis, neuron-glial communication, neuroinflammation, and apolipoprotein E were first chosen, and searches conducted in PubMed, Google Scholar, and Web of Science. The results of these searches were then refined and categorized into cellular contributors at the early stage and later stage of neurodegeneration, based on the characterized Braak-like spatiotemporal staging scheme for tau pathology. Lastly, the combined keywords search strategy was used for searching for potential treatments for tauopathies such as using the affected signaling pathway and tau phosphorylation together. The pathological roles of phosphorylation sites, Hsp90 and site-specific mutations in tau aggregation, the roles of CX3CR1/fractalkine signaling in microglia and neurons, the roles of the glutamate-glutamine cycle between astrocyte and neurons in the progression of tau pathologies, and the possible therapeutic role of NLR3 inflammasome in the treatment of tauopathies are the major focus of this review. A list of the abbreviations used in this review is provided in Table 1.

## 2. Factors Involved in the Formation of Tau Seeds

**Figure 1:**
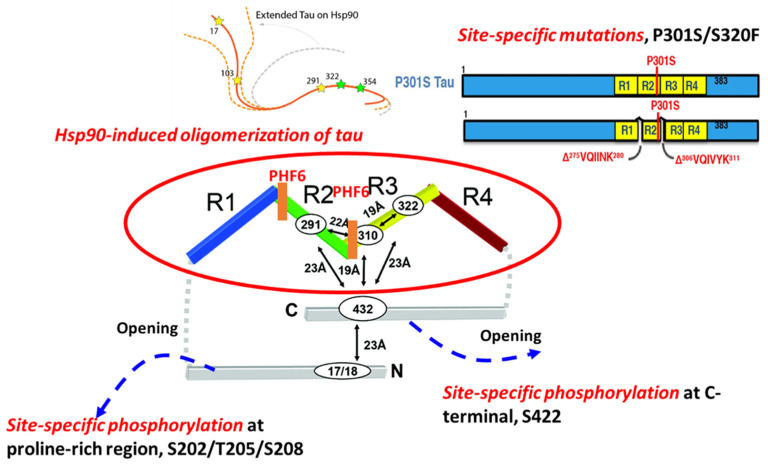
The molecular mechanisms involved in tau aggregation. Molecular factors such as site-specific phosphorylation, site-specific mutations on MAPT, and specific chaperones (Hsp90) are associated with tau aggregation.

The formation of NFTs from soluble tau is a multistep process. This process begins with the dimerization of two conformationally altered monomers and is followed by the formation of intermediate soluble oligomers with varying higher-order conformations and degrees of phosphorylation. Tau oligomers have been implicated as toxic ‘tau seeds’ capable of seeding new aggregates by recruiting normal monomers. Despite evidence of tau trimers being the minimal unit of spontaneous cellular uptake and intracellular fibrillary structure formation in vivo [17], the folding potency of monomer could be much more critical in initiating the early nucleation process of tau aggregation (Figure 1).

### 2.1. Site-Specific Phosphorylation-Mediated Tau Self-Aggregation

Previous work details that although tau itself is intrinsically disordered, proteins in solution possess a ‘paperclip-like’ conformation where the N- and C-terminal ends of tau fold over in proximity to the center of the repeat domains [18]. Site-specific phosphorylation directly influences the conformation of monomeric tau and affects the stability of a folded conformation, contributing to the propensity for tau to aggregate [19]. Two hexapeptides, known as PHF6s, ^275^VQIINK^280^, and ^306^VQIVYK^311^, are located at the beginning of the second and third repeat domains of the MBDs, and appear to drive β-sheet structure formation during the tau aggregation process. The accessibility of residues in the two PHF6s defines the structural differences between inert (Mi) and seed-competent (Ms) tau monomer, meaning that the inert (Mi) tau monomer has less inter-chain accessibility to these residues compared with that in the seed-competent (Ms) monomer [2]. Phosphorylation outside of, but proximal to, these regions is relevant to the formation of NFTs. A previous study systematically investigated the effects of different phosphorylation sites on tau self-aggregation, using a series of in vitro pseudo-phosphorylated tau proteins [20]. Phosphorylation sites T175/T176/T181 within N-terminal, recognized by AT270 antibody, mainly suppress tau aggregation [21]. In addition, phosphorylation at three sites, S202/T205/S208, within the proline-rich region (PRR) is enough to induce tau self-aggregation without any exogenous aggregation inducer [22]. The monoclonal antibody AT8 that specifically recognizes tau phosphorylation at the S202/T205 site has been established as a valid biochemical marker for identifying abnormally phosphorylated tau as well as the paired helical filament form. Moreover, phosphorylation sites near the C-terminus have been found to preferentially promote tau self-aggregation. For example, pseudo-phosphorylated S396, specifically recognized by PHF-1 antibody [21], has led to increased tau aggregation in the presence of metal ion inducer. In particular, the strong effect on aggregation has been seen in pS422 tau protein, which showed increased aggregation in the presence of both metal ions and heparin inducers [20], which may be related to the conversion of tau monomer from inert to seed-competent form, due to increased accessibility of these residues [3], as shown in Figure 1. By performing a comprehensive electrochemiluminescence ELISA assay, Ercan-Herbst et al. [23] found that specific phosphorylation events (pS198, pS199, and pS416) correlated with increased oligomerization in all brain regions, which implies that phospho-sites regulate tau aggregation during the progression of AD neurodegeneration. Collectively, phosphorylation plays a major role in tau self-aggregation by altering the charge and conformations of physiological tau.

### 2.2. Hsp90-Mediated Tau Aggregation

Tau phosphorylation and aggregation that lead to conformational changes could involve molecular chaperones, which regulate protein folding, degradation, and accumulation. The protective effect of Hsp70 and Hsp104 in tauopathies has been described in previous studies [24]. Hsp70 inhibits the aggregation of tau protein by forming a complex with tau oligomer or fibril tau, preventing toxic effects or further seeding of tau aggregation [25,26]. Despite the recognition of its disaggregase activity for many aggregates, a distinct mechanism of Hsp104 in preventing tau aggregation is related to its holdase activity on soluble amyloid tau through the small subdomain of nucleotide-binding domain 2 (ssNBD2) [27].

In contrast to the preventative functions of Hsp70 and Hsp104, heat shock protein 90 (Hsp90), one of the major tau-binding chaperones, has been found to drive the aggregation of tau species [28]. Although Hsp90 is normally thought to act as cellular protection during stress, Hsp90 binding to tau at the VQIVYK motif facilitates a conformational change that results in its phosphorylation by glycogen synthase kinase 3, which further promotes tau aggregation [28]. Additionally, a recent study found that Hsp90 binding to tau uncovered the repeat domains by conformationally opening the ‘paper-clip’ structure of tau, suggesting that the formation of tau oligomers was caused by the conversion of tau monomers from inert to aggregation-prone forms [29].

### 2.3. Site-Specific Mutations and Tau Aggregation

Abnormal tau mutants related to FTDP-17 possess distinct structures leading to a differential aggregation propensity [30,31,32]. Recently, Strang and coworkers demonstrated that the susceptibility of FTDP-17-associated mutants to aggregate with seeded, exogenous fibrillar tau depended highly on site-specific mutations and their surrounding amino acid sequences [33]. Robust aggregation with exogenous tau fibril seeds, both homotypic and heterotypic, has been seen in FTDP-17 mutations at sites P301 and S320. In particular, the unique property of the P301L variant in regulating the aggregation propensity of tau has been demonstrated by mutating individual proline residues into leucine residues within conserved PGGG motifs in each of the four MTBDs in tau [33]. Only P301L showed a propensity to aggregate when seeded with exogenous fibrillar tau. In contrast, other FTDP-17-associated variants near the PHF6 site showed no propensity to aggregate when seeded. Double mutants at P301L/S320F and P301S/S320F have been shown to facilitate aggregation. For these P301L/S320F and P301S/S320F tau protein variants, robust aggregation was observed in vivo without exogenous fibrillar tau seeding [33]. A possible underlying cause of this enhanced aggregation propensity is altered conformation with higher accessibility to PHF6, that converts the inert monomer into aggregation-prone monomer; alternatively, more frequent interactions with chaperones may be required to stabilize a folded conformation for these variants. In either case, further investigation is needed to identify the mechanism.

## 3. Molecular Mechanisms of Tau Cellular Uptake

Transcellular tau propagation has been implicated in tauopathies following a ‘prion-like’ transmission pattern [34], suggesting that the internalization of extracellular tau by recipient cells is mediated mainly by endocytosis. Recent studies showing distinct features of prion-like propagation of tau species under diverse cell and animal models are summarized in Table 2. Endocytosis can be divided generally into clathrin-dependent and -independent internalization, of which the latter can be further divided into caveolin-dependent, -independent endocytosis, and actin-dependent macropinocytosis. Previous studies highlighted cellular internalization pathways associated with tau including bulk endocytosis [35], heparin sulfate proteoglycan (HSPG)-associated macropinocytosis [8], and clathrin-mediated endocytosis [35].

The majority of extracellular tau consists of soluble oligomers and monomers, while a minority of tau species exist in truncated forms cleaved by various proinflammatory cytokines in AD brains [6]. The size and conformation of tau species determine the cellular mechanisms for extracellular tau uptake, which may not be restricted to one particular pathway [8,36]. For instance, smaller sized tau aggregates enter neurons in a dynamin-dependent endocytosis pathway that is independent of actin polymerization [35]. For larger tau aggregates, actin-dependent macropinocytosis has been identified as the main pathway for internalization by neuronal cells [37]. However, the cellular entry pathways of monomeric tau are highly dependent on the specific conformation and isoform. A recent study demonstrated that monomeric tau could enter human neurons via both the dynamin-dependent endocytosis process and through actin-dependent macropinocytosis, which could be regulated by HSPGs [35].

### 3.1. The Effects of HSPGs on the Cellular Uptake of Tau Seeds

HSPGs are highly expressed on the cell surface and have been identified as critical cell-surface endocytosis receptors for tau internalization in various studies. Most recent research has focused on understanding the interaction of heparan sulfate (HS) with tau protein at the structural level, which would provide a mechanistic understanding of how tau-HS interaction regulates tau internalization during the progression of tau pathologies. HS-tau interactions appear to be driven mainly by electrostatic forces between negatively charged sulfo groups on HS and positively charged lysines or arginines on tau protein [38]. Even though electrostatic interactions between tau and HS are relatively nonspecific, a few studies have also identified the importance of specific HS sulfation patterns on the tau-HS interaction. Prior works demonstrated the crucial role of the 6-O-sulfation of HSPGs in the tau-HS interaction by performing an SPR competition assay [39]. Moreover, 6-O-desulfated heparin showed the weakest competitive effect on tau binding to heparin immobilized on a chip among a variety of HS derivatives tested, including N-desulfated and 2-O-desulfated HS derivatives. NMR mapping showed that HS derivatives bound the second repeat motif (R2) in tau. Consistently, a knockout of 6-O-sulfotransferase also significantly reduced tau uptake by HEK293 cells [40]. Reduced intracellular tau uptake and tau cell surface binding in a 3-O-sulfotransferase knockout cell line compared with the wild-type cells suggest that tau protein is capable of recognizing the less common 3-O-sulfation site of HS [41]. The importance of sulfation was further validated in competition assays performed by Zhao and coworkers—3-O-sulfated low molecular weight HS (LMWHS) oligosaccharides had higher inhibitory effects on the tau-HS interaction compared with those without sulfation in an SPR competition assay, further validating the specific role of 3-O-sulfation in tau-heparin interactions. Furthermore, 3-O-sulfation is rare and minor sulfation is found on HS chains, which is likely not responsible for any charge effects in HS chains. More likely, HS interacts with tau via a specific 3-O-sulfation of HS recognized by both the PRR2 and R2 regions of tau instead of non-specific electrostatic interactions. The tau-heparin interaction has also been found to be chain size-dependent due to enhanced electrostatic interactions [40]. Knockouts of extension enzymes of the HSPG biosynthetic pathway, such as extension enzymes exostosin 1 (*EXT1*), exostosin 2 (*EXT2*), and exostosin-like 3 (*EXTL3*) in HEK293T cells significantly reduced the uptake of tau oligomers [36].

HSPGs can be considered as the natural receptors for the uptake of macromolecules, such as larger tau fibrils, through the micropinocytosis pathway; nevertheless, the exact role of HSPGs in the uptake of tau species dominated by the clathrin-mediated pathway needs further investigation within specific systems. Key questions include whether HSPGs are part of a multi-receptor complex or merely an initial attachment site during tau uptake. Moreover, HS-modifying enzyme expression patterns show cell-type-specific patterns, resulting in enormous HS diversity because of the many different cell types in the brain. Because of the heterogeneity of HS-expression, the specific role of HSPGs in tau internalization should be investigated on a cell type-specific basis.

**Table 2:** A summary of recent tau transmission models.

Forms of Tau	Animal/Cell Model	Conformation/Characteristics	‘Prion-Like’ Transmission	Reference
2N4R tau monomer	Mouse primary hippocampal and cortical neurons Non-neuronal cells including HEK293, Hela, MC17, and MLECs	Wild-type tau-paperclip like	Internalized by non-neuronal cells, MLECs 3-O-sulfation is required for HSPG-mediated tau monomer internalization	[41,42]
2N4R tau oligomers		Spherical structure Diameters ranging from 10 nm to 30 nm	Dynamin-dependent endocytosis Bulk endocytosis Tau species in endosome transported in neurons anterogradely and retrogradely	
Heparin-induced tau short fibrils (2N4R)		Helical twist Varied length, from 40 nm to 250 nm		
Tau filaments purified from rTg4510 mouse brain	Primary neurons Hela cells P301L tau expressing mice	Fibrillary structure	Internalized by neuronal cells, but not non-neuronal cells 1-week post-injection, NFTs were detected by immunostaining against MC1at the tau aggregates injection site	
Heparin-induced tau long fibrils (2N4R)	Primary neurons Hela cells	Helical twist Varied length, from 200 nm to 1600 nm	No internalization detected in both neuronal cells and non-neuronal cells	
2N4R tau monomer	Human neuroglioma, H4 cells Human SH-SY5Y hiPSC-derived neurons	Size averaged at 5.7 nm	Internalized by neuronal cells rapidly HSPG-dependent internalization Tau monomer interacts with 6-O-sulfation on the HSPGs, but not N-sulfation	[36]
Heparin-induced oligomer (2N4R)		Helical filaments/19 nm		
Short fibrils (2N4R)		Helical fragments/33nm		
Heparin-induced fibrils (2N4R)		Helical filaments/80 nm	Less internalization detected in both cell lines	
Tau RD monomers	Mouse NPCs, C17.2 Primary neurons	Wild-type tau repeat domain	No internalization detected	[8,40]
Heparin-induced tau fibrils (both RD and WT 2N4R)		Fibrillary structure	Tau fibrils require HSPGs for neurons 6-O- and N-sulfation are required for tau aggregates internalization HSPG mediated intracellular tau seeding Actin-dependent macropinocytosis Clathrin- or dynamin-independent endocytosis	
Tau P301S monomer	hiPSCs-derived cortical neurons	Seeding-prone form	Actin-dependent macropinocytosis Dynamin-dependent endocytosis	[35]
Heparin-induced tau fibrils (Tau P301S)		Fibrillary structure	Dynamin-dependent endocytosis Actin-independent endocytosis	
Tau oligomers	Mouse primary neuronal cells HEK293 CHO cells	Spherical structure	Muscarinic receptor-mediated endocytosis of tau by neuronal and HEK293 cells, but not by CHO cells Muscarinic receptor highly expressed by neuronal cells	[43]
Soluble tau oligomers derived from AD patient	Mouse primary neuronal cells	Spherical structure	Muscarinic receptor-mediated endocytosis of tau	
2N4R tau monomer, oligomers, and fibrils	H4 neuroglioma hiPSCs-derived neuronal cells	Spherical structure Filaments	Receptor-mediated tau endocytosis Knockdown of *LRP1* blocks soluble tau uptake including tau monomers/oligomers, but only reduce tau fibrils uptake	[36]

### 3.2. Receptor-Mediated Endocytosis of Tau

**Figure 2:**
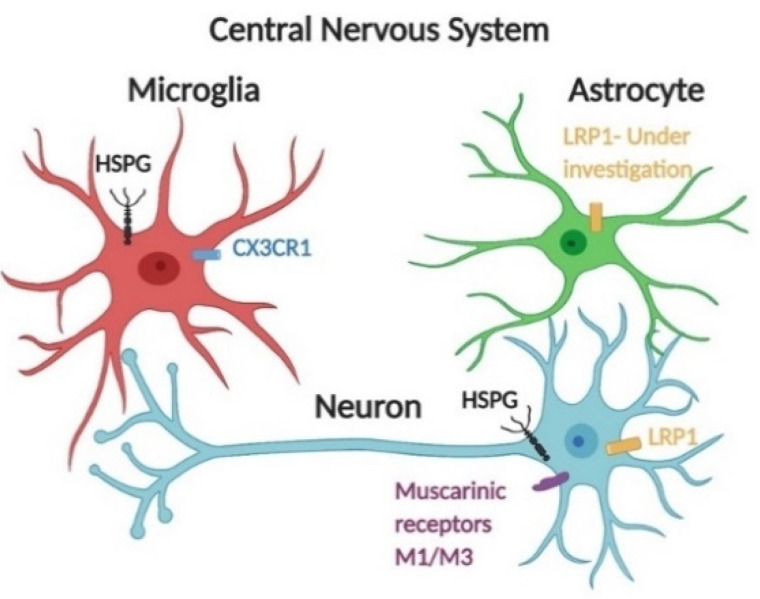
Receptor-mediated endocytosis of tau species is facilitated by several receptors. Central nervous system cells actively internalize monomeric tau via receptor-mediated endocytosis in addition to the HSPG-dependent pathway. Monomeric tau is internalized by muscarinic receptors M1 and M3 in neurons [43]. CX3CR1 mediates monomeric tau uptake in microglia [45]. For astrocytes, monomeric tau can be internalized in a non-HSPG dependent pathway [46]. Further work should be focused on identifying specific receptors of tau endocytosis. Additionally, LRP1 has recently been identified as a major regulator of tau spread in the brain [44]; LRP1 is abundantly expressed by microglia, astrocytes, and neuronal cells [47,48].

Apart from HSPG-dependent uptake, cellular internalization of tau is also regulated by specific receptor-mediated endocytosis, as suggested by several previous studies [35,44]. Rapid dynamin-dependent endocytosis of tau species would typically require one or more receptors, the identities of which are still under investigation. Muscarinic receptors M1/M3 have been found to regulate monomeric tau internalization by neurons [43]. Glial cells including microglia and astrocytes also take up tau efficiently. CX3CR1 has been demonstrated to mediate monomeric tau uptake in microglia [45]. For astrocytes, monomeric tau was internalized in a non-HSPG dependent pathway [46]; further study is still needed to identify specific receptors responsible for rapid dynamin-dependent endocytosis of monomeric tau in astrocytes (Figure 2). Low-density lipoprotein receptor-related protein-1 (LRP1) represents a promiscuous endocytosis receptor for macromolecular ligands, including ApoE and Aβ, and delivers these ligands to the endosomal/lysosomal compartments. Knockdown of LRP1 abolished uptake of various forms of tau, including monomers, oligomers, and fibrils in H4 neuroglioma cells, suggesting that it may serve as a master regulator of tau uptake [44]. Additionally, knocking down LRP1 also prevented tau transmission within human tau transgenic mice. Once associated with specific ligands, LRP1 is also involved in the activation of signaling pathways including MAPK, by assisting the assembly of the intracellular protein complex [47]. LRP1 is also abundantly expressed by radial glia, microglia, and astrocytes, and involved in the clearance of Aβ [47,48]. Further studies are still needed to identify whether and how LRP1 is involved in tau endocytosis by glial cells, and whether the tau-LRP1 interaction alters the immune response of glial cells.

## 4. Cellular Contributors to Tau Pathology

**Figure 3:**
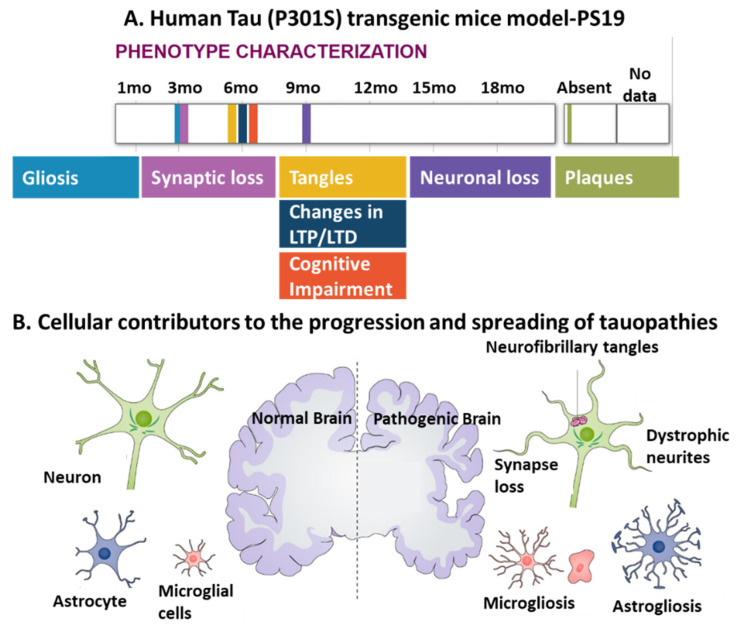
The interplay between different cell types including neurons, microglia, and astrocytes, in tauopathy progression. (**A**) A representative human tau pathology model: PS19 transgenic mice express mutant human *MAPT* with a P301S mutation and display a series of pathological features of tauopathies, such as gliosis, synaptic loss, tangles, and neuronal loss over their lifetimes. Adapted from [51,52]. Note that in this model, no plaques were found; LTP, long-term potentiation; LTD, long-term depression. (**B**) Both microgliosis and astrogliosis are involved in the progression of tauopathies and abnormal neuronal activities, as indicated by phenotypic characterization of human tau pathology models [53]. Copyright © 2021, John Wiley and Sons.

In 1991, the work of Braak proposed the sequence of progression of Alzheimer’s disease neuropathology, demonstrating that soluble hyperphosphorylated tau first appears in the locus coeruleus (LC) neurons and subsequently appears along LC axons to their terminals in the entorhinal cortex (EC) [49,50]. Transgenic mice models that display human tau pathology have been established to recapitulate the development of neurodegeneration and diverse pathological phenotypes, including gliosis, synaptic loss, tangles, and neuronal loss (Figure 3A). These models also demonstrate the involvement of diverse cellular contributors, including neuronal cells, microglia, and astrocytes, to the progression and spread of tauopathies (Figure 3B).

**Figure 4:**
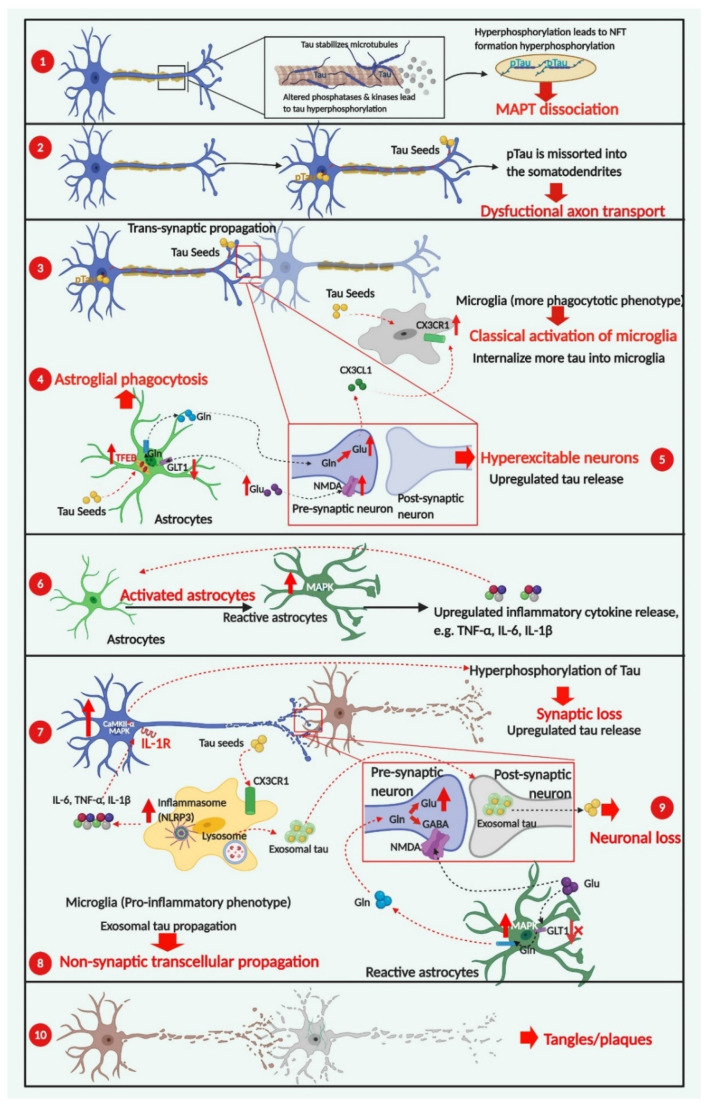
Cellular contributors to tau-dependent degeneration. The development of tau-related pathogenesis has characteristic stages, starting from the formation of tau species consisting of phosphorylated tau dissociated from microtubules ①. Abbreviation: pTau, phosphorylated tau. Hyperphosphorylation of tau incorrectly sorts tau into the somatodendritic compartment, which is linked to dysfunctional axonal transport ②, one of the earliest pathological features of tauopathies. Glial cells adapt a more neuroprotective phenotype with microglia classically activated ③ and astrocytes actively phagocytosing tau species ④. As disease progresses, astrocytes transform into a loss-of-function phenotype via lower-level expression of astrocyte-specific transporters, leading to neuronal excitotoxicity and upregulated tau release. Additionally, alternatively activated microglia in a pro-inflammatory phenotype are necessary and sufficient to induce reactive astrocytes with the capability of releasing neuroinflammatory cytokines ⑥. At the later stages of disease progression, the microglial-exosomal pathway acts as the essential tau propagation pathway ⑧ as an alternative to transsynaptic transduction, due to extensive synaptic degeneration and neuronal death ⑨. The formation of neuronal and glial tau plaques is the most important hallmark of tauopathies ⑩. Red arrows indicate pathological consequences of change. Created in BioRender.com.

As described in detail in the following sections, the development of tau-related pathologies has been postulated to follow spatiotemporal patterns and is characterized by multiple progressive stages, each with pathological features in the form of differential cellular behaviors and distinguished phenotypes (Figure 4). At the earliest stage, tau seeds formed by phosphorylated tau dissociate from microtubules spread along a transsynaptic pathway, involving the release of tau species in the synaptic cleft, with subsequent internalization by post-synaptic neurons [54]. Glial cells, on the other hand, adapt a neuroprotective phenotype with microglia classically activated to engulf tau species in a CX3CR1-dependent way [55], and astrocytes actively involved in clearing tau species with an exacerbated autophagy-lysosomal pathway (ALP) [56]. As the disease progresses, astrocytes display a ‘loss-of-function’ phenotype by exhibiting a decreased level of glutamate transporters, leading to neuronal excitotoxicity and upregulated tau release [57]. Additionally, microglia develop an alternative pro-inflammatory phenotype after responding to diverse pro-inflammatory stimuli, including higher concentrations of tau protein and the presence of reactive oxygen species [11]. These activated microglia continue to produce proinflammatory cytokines, such as TNF-α and IL-1β, which are necessary and sufficient to convert inactive astrocytes into reactive astrocytes, resulting in further neuroinflammatory cytokine release [58].

Activation of the NLRP3 inflammasome in microglia has been demonstrated to facilitate the progression of tau pathologies, mainly through intensifying neuronal tau hyperphosphorylation in an IL-1 receptor-dependent way [59]. At the late stage of disease progression, dysfunctional synaptic transmission caused by synaptic loss and neuronal death leads to microglia-exosomal tau transmission that takes precedence over transsynaptic tau transmission [60].Finally, neuronal and glial tau plaques are formed, which are the most important hallmarks of tauopathies [9].

Overexpression of the ε4 allele of apolipoprotein (*ApoE4*) in multiple cell types shows cell-type-specific effects; overexpression in neuronal cells upregulates neurotransmitter release while enhancing inflammatory signaling of microglia. For astrocytes, ApoE4 overexpression downregulates phagocytosis of pathogenic proteins and disrupts lipid transport and metabolism. Taken together, ApoE4 serves as a common genetic risk in AD and primary tauopathies, and can worsen tau pathology, indicating an overlap between ApoE4 and tau pathogenesis. LRP1, as a major receptor of tau species and ApoE, may play an intermediate role between ApoE and tau species, which could point to a therapeutic potential for treating tauopathies via LRP1 interaction.

### 4.1. The Involvement of Neuronal Activity in the Spreading of Pathogenic Tau

Under physiological conditions, tau is crucial for microtubule stabilization and is located mainly in axons [61]. Immunoblot analysis with phosphorylation-dependent antibodies revealed that phosphorylated tau is missorted into the somatodendritic compartment during the early stages of AD progression [62]. Missorted tau results in axonal transport deficits and loss of synaptic functions and is more prone to forming toxic tau oligomers if seeded [62].

The progression of tau pathology follows a defined hierarchical pattern, starting from the EC, then advancing into anatomically connected neurons downstream in the synaptic circuit, such as the dentate gyrus (DG), the hippocampus, and the neocortex, as demonstrated by tau transgenic animal models [54,63]. Despite the identification of the physiological role of neurons in regulating synaptic tau release and translocation [5], the specific neuronal activities resulting in the propagation of tau pathologies are still under investigation. Amyloid precursor protein (*APP*) transgenic mouse models show that endogenous tau in CSF increases during the progression of amyloid plaque formation, accompanied by hyperexcitable neurons [64,65]. A key question is whether the hyperexcitable neurons are essential for the release of pathogenic tau, independent of Aβ. Indeed, tau pathology mouse models combined with novel neuronal stimulation approaches showed that neuronal hyperexcitability and accelerated synaptic tau release are critically linked and independent of Aβ toxicity [66]. Using an optogenetic activation approach, the stimulated side of the hippocampus of the rTg4510 mice line tended to accumulate more human tau protein, along with increased evidence of neuronal atrophy [66]. Additionally, tau pathology spread from the stimulated-EC to the synaptically connected DG region, suggesting that the propagation of tau pathology accelerates through synaptic circuits.

Abnormal extracellular glutamate levels have been proposed as one of several mechanisms that account for an excitotoxic microenvironment in AD [67]. Notably, alterations in synaptic glutamate homeostasis caused by dysfunctional astrocytes can be deleterious to neuronal cells. To some extent, the activities of reactive astrocytes correlate with the reduction in astroglial glutamate transporters, which in turn elevates the extracellular glutamate level. Accumulation of excess glutamate contributes to neuronal excitability through activating NMDA (N-methyl-D-aspartate) receptors. NMDA receptors, present in glutamatergic neurons, respond to the glutamate levels via binding to their GluN2 subunit that activates increased calcium flux in the neurons [57]. Sequentially, activation of extrasynaptic NMDA receptors has been linked to tau-induced neuronal cell death mediated by calpain I and ERK/MAPK activation [68]. Therefore, alteration of astroglial glutamate transporters and overstimulation of extrasynaptic NMDA receptors of neuronal cells may have an overlapping role in neuronal hyperexcitability, and these actions have been implicated in the progression of tau pathology along with synaptic connections [69].

### 4.2. Glial Cells Are Involved in the Progression of Tau Pathology

Even though tau is expressed primarily by neurons, most primary tauopathies are characterized by the presence of both neuronal and glial tau pathologies [12]. Glial cells adopt immune functions and closely interact with neuronal cells for maintaining brain development and homeostasis [70]. Most glial tau pathologies have been observed in astrocytes and oligodendrocytes, and in some cases, tau pathologies have also been seen in microglia. Moreover, both primary tauopathies and AD are characterized by microgliosis and astrogliosis, along with a significant increase in the pro-inflammatory cytokines [6,71]. Glial cell dysfunction has also been implicated in the progression of neurodegenerative diseases [51]. This part of the review aims to highlight the role of dysfunctional neuronal-glial communication in the spreading and propagation of pathological tau during the progression of tauopathies.

#### 4.2.1. Microgliosis in Tauopathies

##### Neuroprotective Effects of CX3CL1/CX3CR1 Signaling

Microglia are the innate immune cells of the CNS and account for 5–20% of total neural cells in the functional tissue of the brain [72,73]. They have two main CNS functions: immune defense and maintenance and promoting programmed cell death during development [72,73]. Recently, microglia-induced neuroinflammation has been linked to tau hyperphosphorylation, suggesting that microglia play an important role in the progression of tau-related neuropathogenesis [74]. As discussed previously, extensive studies have demonstrated that tau pathology predominantly spreads along with synaptic connections. Physiologically, microglia control and regulate synaptic plasticity through pruning of inactive synapses via phagocytosis during CNS development [75]. Among the key factors emerging as potential regulators of neuronal-microglial interaction, chemokine ligand 1 (CX3CL1) secreted by neurons plays an essential role in regulating phagocytic capability of microglia by binding to CX3CR1 [76], a key receptor that maintains the normal synaptic pruning ability of microglia [77]. Altered CX3CL1/CX3CR1 signaling has been demonstrated to regulate the pathological changes in both animal models of tauopathies and AD patients [78,79]. Single-cell RNA-seq of microglia in AD-transgenic mouse brains shows that CX3CR1 is upregulated as part of the initial innate immune response [80], which facilitates the internalization of tau by microglia to enhance the clearance of extracellular Tau [55].

However, at the later stages of AD, CX3CR1, among many other genes, is downregulated [80]. The downregulation of CX3CR1 has also been observed in human brain tissue from AD patients, showing that CX3CR1 levels decrease as microglial phagocytic phenotypes are reduced [55]. Microglia have been found to phagocytose extracellular tau oligomers directly via the tau-CX3CR1 interaction, which is impaired by the loss of CX3CR1 at the later stages of AD. The deletion of CX3CR1 in models of tau pathology has accelerated tau phosphorylation and exacerbated neurodegeneration [55,81]. This CX3CR1 deficiency led to elevated levels of tau phosphorylation on the AT8 (pS202), AT180 (pT231), and PHF1 (pS396/S404) epitopes [58], which is mediated by neuronal IL-1 and TLR-4 receptors triggered by the microglial release of proinflammatory cytokines [82]. Indeed, the deletion of CX3CR1 in the hAPP-transgenic mice model exacerbates microglial inflammation and neurotoxicity by upregulating the secretion of proinflammatory cytokines [78]. Similarly, CX3CL1 overexpression in the human tau transgenic mouse model rTg4510 significantly reduced neurodegeneration and microglial activation [83]. Therefore, the investigation of CX3CR1-CX3CL1 signaling has provided novel insights for treating tauopathies.

##### The Role of NLRP3-ASC Inflammasome Activation in Tau Phosphorylation

Extracellular fibrillary Aβ-induced microgliosis has been linked to NOD-like receptor family, pyrin domain containing 3 (NLRP3) inflammasome activation, which further exacerbates Aβ pathology [84]. The role of microglia and NLRP3-caspase recruitment domain (ASC) inflammasome activation has been demonstrated recently in Aβ-independent tau pathology [59]. Phagocytosis of fibrillar Aβ induced the assembly of the NLRP3 inflammasome consisting of NLRP3, ASC, and pro-caspase 1, which led to the caspase 1-dependent release of pro-inflammatory cytokines such as IL-1β and IL-18 [85]. Stancu and colleagues [86] demonstrated that aggregated tau was capable of activating the NLRP3 inflammasome, which further exacerbated the tau aggregate seeding and increased the secretion of proinflammatory cytokines. The importance of NLRP3 on the progression of tau pathology also was demonstrated in tau transgenic mice models deficient for NLRP3 or ASC. A significantly lower level of tau phosphorylation was observed in the hippocampal samples of the transgenic mice deficient for NLRP3 or ASC compared with wild-type mice [86]. Additionally, templated seeding of tau pathology was reduced in tau transgenic mice with an ASC deficiency.

**Figure 5:**
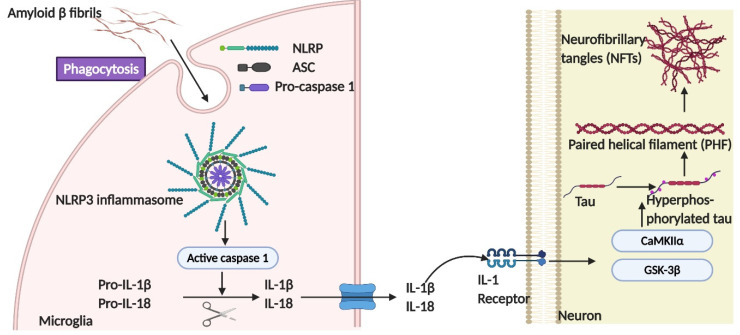
The role of the NLRP3-ASC inflammasome in tau pathogenesis. Either fibrillary Aβ or tau species in the form of monomers or oligomers are sufficient to induce the assembly of the NLRP3 inflammasome, consisting of NLRP3, ASC, and pro-caspase 1, which further leads to caspase 1-dependent release of the pro-inflammatory cytokines IL-1β and IL-18 [85]. The activation of the NLRP3 inflammasome in microglia has been demonstrated to promote neuronal tau hyperphosphorylation in an IL-1 receptor-dependent manner via the regulation of multiple tau kinases, like GSK-3β and CaMKII-α.

Reduced activities of GSK-3β and CaMKII-α, but not p38/MAPK and Cdk5, were correlated with the deficiency of NLRP3 or ASC, suggesting the potential role of the NLRP3 inflammasome in regulating tau kinases in neuronal cells [86]. To understand how the NLRP3 inflammasome regulates tau phosphorylation, conditioned medium collected from LPS-activated microglia induced an increased level of tau phosphorylation in neuronal cells, along with the activation of CaMKII-α [59]. However, once the neuronal IL-1 receptor was inhibited, the effects on CaMKII-α were abolished, suggesting that the activation of the NLRP3 inflammasome in microglia promotes neuronal tau hyperphosphorylation in an IL-1 receptor-dependent manner via the regulation of multiple tau kinases (Figure 5). Potential therapeutic interventions targeting the NLRP3 inflammasome have been attempted for treating AD in mouse models [87]. By increasing *ASC* and *NLRP3* gene expression in Tau22 transgenic mice, the formation of tau aggregates was attenuated, as determined by thioflavin T staining and reduced tau phosphorylation at serine 416, due to diminished CaMKIIα activity [87]. Pharmacological NLRP3 inhibition using the molecular inhibitor MCC50 also significantly decreased tau-seed induced tau aggregates, as determined by AT8 detection, in tau transgenic mice [86].

Even though these studies demonstrate the involvement of neuroinflammation and altered CX3CR1-CX3CL1 signaling in the spreading of tau pathology, further investigation is still needed to uncover the interplay between neuroinflammation induced by extracellular tau aggregates and disrupted phagocytosis caused by impaired CX3CR1-CX3CL1 signaling. Most likely, the relationship between inflammation and phagocytosis will demonstrate the crucial role of microglia in the development of tau pathology.

##### Non-Transsynaptic Tau Propagation-Microglial and Exosomal Spreading of Tau Species as an Alternative Pathway

In addition to the important role of CX3CR1-CX3CL1 signaling and the NLRP3 inflammasome in tau pathogenesis via the crosstalk between neurons and microglia, exosomes are another key mediator between glial-neuronal communication for both synaptic pruning in the healthy brain as well as neuroinflammation under pathological conditions [88,89]. Physiologically, neuronal exosomes stimulate microglial phagocytosis under selective elimination of synaptic connections. Microglia-derived exosomes play a major role in hierarchical tau transmission [60], despite pathogenic tau readily propagating from neuron to neuron in the form of free-floating fibrils [34] and interconnected neuronal contacts [90], as well as neuronal exosomes [4].

Recently, a tau rapid-propagation mouse model was created with adeno-associated viral (AAV)-tau injection into the EC [60]. This model exhibits rapid tau pathology, as demonstrated by the spreading of human tau from EC to the DG within 1 month, recapitulating the perforant diffusion pathway of AD progression in the human brain [91]. Moreover, inhibition of exosome synthesis or depletion of microglia in this AAV-GFP/tau injection mouse model led to a dramatic reduction of AT8+ tau detected in the DG without changing the tau expressed in the injection site, indicating the important role of microglia-derived exosomes in the spreading of tau pathology. Pharmacologic inhibition of exosome synthesis in microglia not only dramatically reduced secretion of the tau-containing exosomes but also decreased the capabilities of exosomes to deliver hTau, as observed in co-cultured primary neurons [60]. As the synaptic connection becomes less functional throughout disease progression, the microglial and exosomal transmission pathways become the primary means of tau propagation [9], suggesting exosomal transmission as a potential therapeutic target.

#### 4.2.2. Astrogliosis in Tauopathies

The concept of astroglial excitability—activation of membrane ion receptors in response to stimulation—facilitates the bidirectional communication between neurons and astrocytes mediated by a ‘tripartite synapse’ [92]. The close physical proximity between synapses and astrocytes and resulting efficient neurotransmission explain why astrocytes are key regulators in maintaining essential neuronal functions, including synaptic plasticity and neurodevelopment [57]. Besides their crucial role in supporting neuronal functions in the CNS, astrocytes represent the largest group of glial cells that interact closely with microglia for maintaining efficient immune surveillance of the CNS [92]. Like microglia, astrocytes also express genes involved in phagocytosis [93], and eliminate synaptic debris [94], and protein aggregates, as seen by the clearance of Aβ [95]. In recent years, the involvement of astrocytes in the progression of tau pathology has drawn much attention because of their widely demonstrated role in the progression of neurodegeneration in tauopathies [11,57]. For example, reactive astrocytes induced by microglial activation have been observed to precede tangle formation in P301S tau transgenic mice models (PS19) [51].

##### Reactive Astrocyte Phagocytosis Has a Neuroprotective Effect

Under pathological conditions, astrocytes develop more neurotoxic features by transforming into reactive astrocytes (A1 subtype), induced by activated microglia and neuroinflammation in various human neurodegenerative disorders [16]. The phagocytic ability of reactive astrocytes appears to be enhanced in tau transgenic mouse models [96]. Astrocyte activation is accompanied by upregulated expression of transcription factor EB (TFEB), the key regulator of the autophagy-lysosomal pathway (ALP). When compared with wild-type counterparts, two widely used tau pathology mouse models, rTg4510 and PS19, showed increased expression of TFEB and lysosomal protein LAMP1 [56]. In particular, astrocytes of rTg4510 mice (transgenic mice expressing human P301L tau protein) showed much higher nuclear localization of TFEB in GFAP-expressing astrocytes compared with the wild-type mice [96]. However, overexpression of astrocytic TFEB in rTg4510 showed minimal effects on neuronal activities. In vitro, TFEB overexpression in primary astrocytes led to enhanced cellular uptake of tau fibrils by stimulating lysosomal biogenesis. In contrast, the TFEB-transduced PS19 tau pathology mouse model showed reduced tau pathogenesis and reduced tau transmission compared to the rTg4510 mouse model. These data demonstrated that the neuroprotective effects of astroglial activation took place primarily at the early stage of tauopathies by enhancing endocytosis and subsequently, triggering intensive lysosomal-mediated degradation of abnormal tau species. The effects of ALP on regulating phagocytic properties of reactive astrocytes may be one of the mechanisms that explains why tau protein enters astrocytes more efficiently than neurons, as observed in prior work [97] and has been implicated in the glial inclusions, as seen in most of the primary tauopathies, including PSP, CBD, and PiD [97].

##### Neuroinflammatory Microenvironment Induced by Reactive Astrocytes

Reactive microglia secrete inflammatory cytokines such as IL-1a, TNF-a, C1q, and IL-1β [16,97]. These cytokines themselves are necessary and sufficient to induce the A1 subtype of astrocytes, which further stimulates inactive astrocytes in proximity. Reactive astrocytes lose their ability to promote neuronal survival, synaptogenesis, and phagocytosis. Enhanced release of inflammatory cytokines from activated glial cells can induce active neuronal p38 MAPK by interacting with multiple receptors, such as TNFR1, and lead to enhanced phosphorylation and aggregation of tau, which precedes the progression of tau pathology [98]. Thus, reactive astrocytes also play a role in neuroinflammation-induced tau pathology.

##### Dysfunctional Neuronal-Astroglial Communication

Reactive astrocytes also exhibit neurotoxicity by impairing glutamate transport between neurons and astrocytes and disturbing the synaptic neurotransmitter balance via direct contact [57,99]. Astrocytes are key regulators for maintaining homeostasis of major neurotransmitters like glutamate (Glu) and γ-aminobutyric acid (GABA) via the glutamine-glutamate/GABA cycle [99]. The rapid uptake of tau species by reactive astrocytes disrupts intracellular Ca^2+^ signaling, leading to a significant reduction in the release of gliotransmitters such as glutamate, glutamine, and serine, and formation of synaptic vesicles [97]. Moreover, it has also been reported that conditioned medium (CM) collected from primary astrocyte cultures isolated from P301S mice decreased the expression of synaptic neuronal markers in cultured cortical neurons, while CM from control astrocytes enhanced these markers in co-cultured neurons [100]. Taken together, reactive astrocytes appear to affect neuronal tau pathologies by impairing the neuronal synaptic transmission as well as synaptic plasticity.

The glutamine (Gln)/glutamate (Glu) cycle (GGC) is critical for maintaining homeostasis of the major neurotransmitters Glu and γ-aminobutyric acid (GABA), which is a key metabolic interaction between neurons and astrocytes [57,101]. Astrocytes uptake excess Glu released by glutamatergic neurons in the synaptic cleft via glutamate transporter-1 (GLT1) receptor (see Figure 4). Glu is then converted into Gln by astrocyte-specific enzymes, and released into the extracellular space. Subsequently, Gln is taken up by neurons and metabolized into Glu by neuron-related enzymes. Recent studies have reported that the dysfunctional neuronal-astroglial communication via the GGC may contribute to tau protein pathology [57]. For instance, reduced expression of astrocytic glutamate transporters, such as GLT1, has been found to coincide with tau inclusion pathology, as well as neuromuscular weakness in the spinal cord and the brainstem, as seen in both tau transgenic mouse models and CBD patients [99]. The reduction of glutamate transporters in astrocytes also elevates extracellular glutamate levels that then further overstimulate glutamatergic receptors (NMDA receptors), causing increased calcium flux and more neuronal excitotoxicity [62]. Subsequently, activation of NMDA receptors has led to tau phosphorylation at specific sites, the most efficient being Ser-396, mediated by p38/MAPK activation [102]. The mechanistic effects of tau pathology on the downregulation of glutamate transporters and reduction of GLT1 in glial cells are unknown, but investigations on the involvement of astrocytes in the progression of tauopathies have provided novel insights for treating glial tau pathology.

### 4.3. ApoE4 Plays a Cell-Type Dependent Role in Tau Pathology

ApoE protein serves as a major cholesterol carrier in the brain, as well as helping to clear Aβ plaques. Among the three alleles for ApoE, the presence of ApoE4 is considered an important genetic risk factor for Alzheimer’s disease, leading to tau hyperphosphorylation in an Aβ-dependent manner [103]. However, a key question is whether ApoE4 influences tau pathology in primary tauopathies, such as PSD, in which tau pathology is not accompanied by Aβ. Using a P301S transgenic mouse model, Shi et al. [104] have demonstrated ApoE4-induced tau pathology independent of Aβ pathology, suggesting the crucial role of cholesterol in the tau pathogenesis of primary tauopathies.

ApoE4 expressed by different cell types has been shown to affect tau pathologies in a cell type-dependent manner. ApoE4 toxicity has been observed in multiple cell types, including neurons, as demonstrated in human iPSCs-derived cell types [105] despite ApoE4 being primarily produced by microglia and astrocytes in the CNS. ApoE4-expressing neurons exhibited tau hyperphosphorylation, while ApoE4 glial cells had reduced capacity for neuronal homeostasis and thus contributed to the pathogenesis of tau pathology. For instance, Wang and colleagues [106] showed that ApoE4-expressing neuronal cultures derived from human iPSCs expressed higher levels of the synaptic proteins SYN1 and PSD95, alongside an elevated release of neurotransmitters, compared with ApoE3-expressing neurons. Additionally, ApoE4 cerebral organoids exhibited an elevated level of phosphorylated tau (p-S202/T205) compared with ApoE3 organoids. ApoE4-expressing astrocytes had impaired lipid metabolism/transport and phagocytosis, while ApoE4-expressing microglia exhibited intensive immune reactivities upon LPS stimulation compared with wild-type microglia.

Neurons co-cultured with ApoE knockout glial cells displayed the greatest neuronal viability with the lowest level of TNF-α secretion [104]. Analogously, co-culturing P301S tau-expressing neurons with ApoE4-expressing microglia resulted in markedly reduced neuronal viability and a significantly high level of TNF-α secretion. Similarly, Friedberg and colleagues have demonstrated that inflammatory profiles of AD-associated microglia that regulate tau pathologies are highly dependent on the presence of ApoE4 [107]. These data have suggested that ApoE4 plays a crucial intermediate role between microglia inflammatory signaling and tau pathology. Furthermore, LRP1, a major receptor for ApoE and tau, has been shown to mediate the inflammatory responses of microglia via the regulation of the JNK and NF-κB signaling pathways [47]. ApoE may facilitate the assembly of the tau-LRP1 complex to exacerbate the pro-inflammatory signaling pathways on microglia.

## 5. Therapeutic Approaches Targeting Molecular/Cellular Signaling Pathways

A comprehensive understanding of cellular and molecular contributors to tau pathogenesis provides novel insights for discovering therapeutics for human tauopathies, including AD. Extensive investigations have demonstrated that HSPGs play a crucial role in the transcellular spreading of tau pathogenesis; therefore, HS-based therapeutics hold great potential for treating tau pathologies. Small molecules or anti-HS peptides interfering with HS-tau interaction are of therapeutic potential for the treatment of tauopathies, which have been reviewed previously [108]. Glycan-based compounds targeting 3-O-sulfated motifs on HS recognized by tau seeds represent a novel therapy for tauopathies [41].

NLRP3 inflammasome activation has been linked to the development of multiple inflammatory diseases, such as atherosclerosis, type II diabetes, and Alzheimer’s disease, as well as various cancers [109]. The inhibition of NLRP3 inflammasome activity has been demonstrated to decrease tau phosphorylation and aggregation via attenuated neuronal GSK-3β and CaMKIIα activities. Antagonizing purinoreceptor (P2 × 7R) to prevent the assembly of an active NLRP3 inflammasome in microglia has been suggested as one of the best approaches to control neuroinflammation caused by microglial activation and has therapeutic potential for treating tauopathies [109,110].

Hsp90 directly binds to tau species [110], and Hsp90 inhibitors have been considered as promising therapeutics for treating tauopathies. However, disappointing clinical results due to poor blood–brain barrier permeability and toxicity of all tested drugs have led researchers to alternatives to Hsp90, such as Hsp90 co-chaperones, including ATPase homolog 1 (Aha1), a small 38-kDa cochaperone that binds to the N-terminal and middle domains of Hsp90. The role of Aha1 in tau pathogenesis via interactions with Hsp90 has been demonstrated in a transgenic tau mouse model, rTg4510 [111]. Overexpression of Aha1 led to an increased level of sarkosyl-insoluble tau, as well as the tau with T22 reactivity (anti-oligomer antibody). Treatment with KU-177, which binds specifically to Aha1, reduced the accumulation of insoluble P301L tau in cultured cells, suggesting that Aha1 may be a promising therapeutic target for tauopathies by directly reducing tau aggregation [111].

The autophagy-lysosomal pathway (ALP) shows beneficial effects on tau clearance in reactive astrocytes during the early stages of tau pathogenesis. Because of this, activation of TFEB, a key regulator of this pathway, could be considered as a promising treatment for tauopathies [96]. The therapeutic role of a novel TFEB activator named curcumin analog C1 has been studied using three AD animal models [56]. Treatment with curcumin analog C1 has significantly reduced the levels of Aβ42/Aβ40 in brain lysates from 5×FAD mice models, and phospho-tau epitopes (AT8+ and PHF1+) in a P301S mice model. In addition, curcumin analog C1 attenuated both APP and tau pathology in a 3×Tg AD mice model, accompanied by TFEB activation, increased autophagy, and lysosomal activity.

Neuroprotective effects of CX3CR1-CX3CL1 signaling in tau clearance through microglia phagocytosis has been suggested at an early stage of tau pathogenesis, revealing that the enhancement of this signaling at an early stage of disease progression could be beneficial for disease treatment. Indeed, soluble CX3CL1 overexpression by adenoviral transformation in the Tg4510 mouse model has rescued tau pathology by regulating microglial activation [83]. An alternative approach by Fan et al. [112] via neuronal CX3CL1 overexpression reduced neuronal loss and improved cognitive function in a P19 tauopathy model by enhancing neurogenesis through the CX3CL1–TGF-β2/3–Smad2 pathway. Taken together, CX3CL1 overexpression could be considered a key therapeutic target for treating AD by either promoting neurogenesis for neuronal loss recovery or attenuating microglia-induced neuroinflammation.

Given the overlapping effects on neuronal excitotoxicity by overstimulation of NMDA receptors and decreased expression of astrocyte-specific glutamate receptors observed in multiple neurodegenerative disorders, both NMDA receptors and reactive astrocytes have been implicated as therapeutic targets for treating tau pathology including AD. NMDA receptor-dependent excitotoxicity has been shown to depend on the extrasynaptic GluN2B-containing NMDA receptors rather than synaptic GluN2A-containing NMDA receptors. Antagonists selectively inhibiting extrasynaptic NMDA receptors may have neuroprotective effects [113]. Recently, a new NMDA receptor blocker, RL-208, has been tested on a mouse model of late-onset AD, showing cognitive function improvement in terms of increased synaptic protein density, increased phosphorylation of NMDA2B, reduced protein-related apoptosis, as well as decreased phosphorylated tau levels [114]. This study points out that this novel neuroprotective drug may be valuable for treating AD.

Ameliorating dysfunctional neuronal-astrocyte communication via reducing reactive astrocytes may pose an additional therapeutic target for neurodegenerative disorders. Decreased levels of astrocyte-specific glutamate transporters have been associated with the pathogenesis of tau pathology [115]. For instance, small molecule LDN/OSU-0212320 has been shown to upregulate the expression of EAAT2, a glutamate transporter, in astrocytes via translational activation. Thus, LDN/OSU-0212320 treatment has attenuated glutamate-induced cytotoxicity in neuron and astrocyte coculture, as indicated by the greatest neuronal survival compared to untreated cells. Furthermore, significantly ameliorated symptoms and prolonged lifespan upon the treatment with LDN/OSU-0212320 have been demonstrated in an ALS transgenic mice model [116]. Similar studies are still warranted to determine the drug efficacy for AD models.

*ApoE4* is the most recognized genetic risk factor of AD and is thus considered an important therapeutic target for treating AD. Despite the incomplete understanding of the mechanisms underlying the effects of *ApoE4*, the conversion of *ApoE4* to less-toxic isoforms of *ApoE*—either *ApoE3* or *ApoE2*—may hold promising therapeutic potential. For instance, gene editing to convert *ApoE4* to *ApoE3* or the addition of a ‘structural corrector’ on *ApoE4*-expressing culture to refold *ApoE4* into more *ApoE3*-like conformation has rescued *ApoE4* neurons from AD pathology [106]. Additionally, an AAV-mediated *ApoE2* expression vector targeting the *ApoE4* gene of AD patients to transform the ApoE4 homozygote to an *ApoE2-ApoE4* heterozygote for treating AD is currently in clinical trials [117]. A summary of therapeutic approaches targeting altered molecular and cellular signaling pathways in tauopathies is presented in Table 3.

## 6. Conclusions

Tauopathies are characterized by multiple pathological features, including the transcellular propagation of pathogenic tau seeds, neuronal loss, neuroinflammation, and neurofibrillary tangles. HSPGs and LRP1 have been identified as major receptors for transneuronal propagation of pathogenic tau. Altered neuronal tau kinases contribute to hyperphosphorylated tau, which is further exacerbated by intracellular oxidative stress, microglial NLRP3 inflammasome activation, and dysfunctional astroglial-dominant glutamate transport and metabolism. Neuronal loss is the consequence of both neuronal excitotoxicity and pathological tau toxicity, mainly manifested by dysfunctional axonal transport, synaptic degeneration, and upregulated levels of pro-inflammatory cytokines. As pathological phenotypes continue to develop, tau inclusions become increasingly evident and severe, which gives further rise to the pathogenic tauopathy diagnosis. The NLRP3 inflammasome, the autophagy-lysosomal pathway, CX3CL1 signaling pathway, and ApoE4 are all therapeutic targets that could yield potential new treatments for tauopathies. Interventions of tau self-aggregation via disease-specific antibodies or Hsp90 inhibitor provide one novel tool for tau pathology at an early stage of neurodegeneration. Because of the neuroprotective effect of the CX3CR1/CX3CL1 signaling pathway between microglial and neuronal communication, CX3CL1 or CX3CR1 overexpression may protect neuronal cells from toxic tau by enhancing neurogenesis, as well as internalizing more soluble tau from the extracellular matrix. As a link between amyloid deposition and neurofibrillary tangle formation, targeting the activation of the NLRP3 inflammasome provides a promising approach for the development of therapies for AD via inhibiting two hallmarks of AD simultaneously. Translational activation of glutamate transporters shows the advantageous effects on rescuing the dysfunctional GGC between astrocyte and neurons, as well as ameliorating the neurotoxicity caused by the high level of extracellular glutamate.

## Figures and Tables

**Table 1:** Table of abbreviations used in this review.

Abbreviation	Explanation
AD	Alzheimer’s disease
MAPT	Microtubule-associated protein tau
ApoE	Apolipoprotein E
NFT	Neurofibrillary tangles
PSP	Supranuclear palsy
CBD	Corticobasal degeneration
PiD	Pick’s disease
FTDP-17	Frontotemporal dementia with Parkinsonism linked to chromosome 17
CNS	Central nervous system
MBD	Microtubule-binding domain
Aβ	Amyloid β
PRR	Proline-rich region
PHF	Paired helical filaments
ISF	Interstitial fluid
CSF	Cerebrospinal fluid
HSPG	Heparan sulfate proteoglycans
ALP	Autophagy-lysosomal pathway
NMDA	N-methyl-D-aspartate
TFEB	Transcription factor EB
LTP	Long-term potentiation
LTD	Long-term depression

**Table 3:** Potential therapeutic targets for the treatment of tauopathies.

Signaling Targets	Therapeutics	Settings/Organisms	Outcomes/Affected Functional Effects	Affected Phosphorylated Sites	State of Process	Reference
Interfering HSPG-tau interaction	HS oligosaccharide containing two 3-O-sulfo group	Mouse lung endothelial cells (MLECs)	Blocking tau cell surface binding and internalization	NA	Research stage	[41]
Intervening the formation of NLRP3 inflammasome	Cias1 and *Pycard* gene dysfunction	FTD mice model, ACS- or NLRP3 -deficient Tau22 mice	Attenuating the level of tau hyperphosphorylation (AT8) in hippocampus Decreasing level of GSK-3β and CaMKII-α activities	pS416	Research stage	[59,87]
Hsp90 cochaperones, Aha1	KU-177	Inducible HEK-P301L cells transfected with Aha1	Reduced insoluble tau	pS396/404	Research stage	[111]
Promoting the autophagy-lysosomal pathway (ALP)	Curcumin analog C1	Transgenic mice models, 5×FAD, P301S, and 3×Tg	Attenuating both APP and tau pathology Activating autophagy-lysosomal pathway (ALP)	pS396/404 pS202/T205	Research stage	[56]
Promoting CX3CL1 regulated signaling	CX3CL1 overexpression	P19 tau pathology mice model	Reducing neurodegeneration and improving cognitive function with increased neurogenesis	NA	Research stage	[112]
Inhibiting extra-synaptic NMDA receptor	RL-208	A mice model of late-onset AD (LOAD)	Reduced apoptosis-related proteins, caspase-3 and calpain-1 Increased phosphorylation level of NMDA2B Decreased kinase activity of Cdk5/p25 accompanied by a reduced level of p-tau	pS396	Research stage	[114]
GLT1 upregulation	LDN/OSU-0212320	Neuron and astrocyte coculture	Upregulating the expression of EAAT2 in primary astrocytes Preventing neurons from glutamate-induced cytotoxicity in a neuronal-astrocytic coculture	NA	Research stage	[116]
ApoE4 gene therapy	AAV ApoE2-expressing vector targeting CNS/CSF ApoE4	AD patients	Targeting to transform ApoE4 in CNS/CSF of AD patients into ApoE2, which is less toxic	NA	Phase I	[117]
